# Metroplasty before IVF in women with a T‐shaped uterus: A clinical challenge

**DOI:** 10.1002/ijgo.70827

**Published:** 2026-01-22

**Authors:** Fabio Barra, Irene Gazzo, Alessandro Favilli, Consuelo Russo, Giulia Monaco, Franco Alessandri, Sandro Gerli, Luis Alonso Pacheco, Caterina Exacoustos, Simone Ferrero

**Affiliations:** ^1^ Unit of Obstetrics and Gynecology IRCCS Ospedale Policlinico San Martino Genoa Italy; ^2^ Department of Health Sciences (DISSAL) University of Genoa Genoa Italy; ^3^ Department of Reproductive Medicine Ospedale Evangelico Internazionale Genoa Italy; ^4^ Section of Obstetrics and Gynecology, Department of Medicine and Surgery University of Perugia Perugia Italy; ^5^ Gynecological Unit, Department of Surgical Sciences University of Rome “Tor Vergata” Rome Italy; ^6^ Unidad de Endoscopia Ginecológica, Centro Gutenberg Hospital Xanit Internacional Málaga Spain; ^7^ Academic Unit of Obstetrics and Gynecology IRCCS Ospedale Policlinico San Martino Genoa Italy; ^8^ Department of Neurosciences, Rehabilitation, Ophthalmology, Genetics, Maternal and Child Health (DiNOGMI) University of Genoa Genoa Italy

**Keywords:** hysteroscopy, in vitro fertilization, infertility, metroplasty, Müllerian anomalies, T‐shaped uterus

## Abstract

A T‐shaped uterus is a uterine malformation, which can be either congenital or acquired, potentially impairing fertility and increasing the risk of miscarriage. Diagnosis primarily relies on three‐dimensional ultrasound and hysteroscopy. Hysteroscopic metroplasty is the standard surgical intervention aimed at restoring normal uterine anatomy and potentially improving reproductive outcomes. This narrative review, conducted in accordance with SANRA (Scale for the Assessment of Narrative Review Articles) guidelines, is based on a comprehensive database search and critically evaluates observational and interventional studies on the definition, diagnosis, and management of T‐shaped uterus in the context of in vitro fertilization. Recent studies indicate that correction of this anomaly might not only improve spontaneous conception rates but also enhance the outcomes of IVF. However, the precise role of metroplasty in patients undergoing IVF outcomes remains controversial. Some evidence indicates that surgical correction might improve embryo implantation and endometrial perfusion. Despite these promising observations, randomized controlled trials are necessary to define optimal patient selection criteria and to confirm the true benefit of metroplasty in the context of IVF. Future research should also address the potential risks associated with the procedure. A standardized diagnostic and therapeutic approach might contribute to improved reproductive outcomes in affected patients.

## 1 INTRODUCTION

Congenital malformations of the female genital tract are anatomical abnormalities resulting from improper embryological development of the Müllerian (paramesonephric) ducts.^1^, ^2^ The T‐shaped uterus, one of the most frequent of these anomalies, has been defined by the European Society of Human Reproduction and Embryology (ESHRE) and the European Society for Gynecological Endoscopy (ESGE) as a condition characterized by a narrow uterine cavity due to thickened lateral walls, with a 2:1 ratio between the uterine corpus (two‐thirds) and the cervix (one‐third). This malformation leads to an endometrial cavity that resembles the shape of the letter “T” rather than the typical triangular configuration. However, no specific criteria or cutoff values for lateral wall thickening or cavity narrowing were provided at that time. According to the ESHRE classification system, which divides uterine anomalies into six categories, the T‐shaped uterus is included under Class U1/a (dysmorphic uterus – T‐shaped), and is differentiated from Class U1/b (infantile uterus) and Class U1/c (other dysmorphic types).^1^

The T‐shaped uterus, once primarily linked to in utero exposure to diethylstilbestrol (DES), a synthetic estrogen employed between 1940 and 1971 to prevent pregnancy complications,^3^, ^4^, ^5^ continues to be diagnosed today, indicating that congenital or acquired causes unrelated to DES also exist.^6^

Although the T‐shaped uterus can be asymptomatic, it has frequently been identified in women with infertility or recurrent pregnancy loss (RPL), raising concerns about its potential impact on reproductive outcomes. Nevertheless, its true prevalence in the general population remains unclear, in part due to discordance in diagnostic criteria and variability in patient populations studied.^7^, ^8^, ^9^

Diagnosis typically relies on imaging techniques such as three‐dimensional transvaginal ultrasound (3D‐TVS), which allows visualization of the uterine cavity in the coronal plane, and hysteroscopy, which can serve both diagnostic and therapeutic purposes.^8^, ^10^

Hysteroscopic metroplasty is the most commonly proposed treatment for a T‐shaped uterus.^10^ In the last 30 years, there has been growing interest in better understanding the role of surgical correction for dysmorphic uteri, particularly in the context of in vitro fertilization (IVF) and repeated implantation failure (RIF). However, robust data from randomized controlled trials are lacking, and clinical decision‐making often relies on observational studies and expert opinion.

In this review, we aim to investigate whether surgical correction of a T‐shaped uterus confers a significant benefit in achieving pregnancy among women undergoing IVF for infertility or RPL.

## 2 METHODS

This narrative review followed the SANRA (Scale for the Assessment of Narrative Review Articles) guidelines to ensure methodological rigor.^11^ A comprehensive search of PubMed, Scopus, and Google Scholar was conducted for English‐language articles up to December 31, 2024, using the terms “T‐shaped uterus,” “dysmorphic uterus,” “in vitro fertilization,” “metroplasty,” and “reproductive medicine,” combined with Boolean operators. Reference lists were also screened for additional studies. Eligible observational and interventional studies addressing definition, pathophysiology, diagnosis, clinical features, risk factors, and management of a T‐shaped uterus in the context of IVF were included. Study selection occurred in three stages (title, abstract, and full text) by two independent reviewers, with disagreements resolved by consensus. Data extraction focused on the most scientifically robust evidence, and potential sources of bias (publication, funding, and conflicts of interest) were critically assessed.

## 3 DIAGNOSIS

**FIGURE 1:**
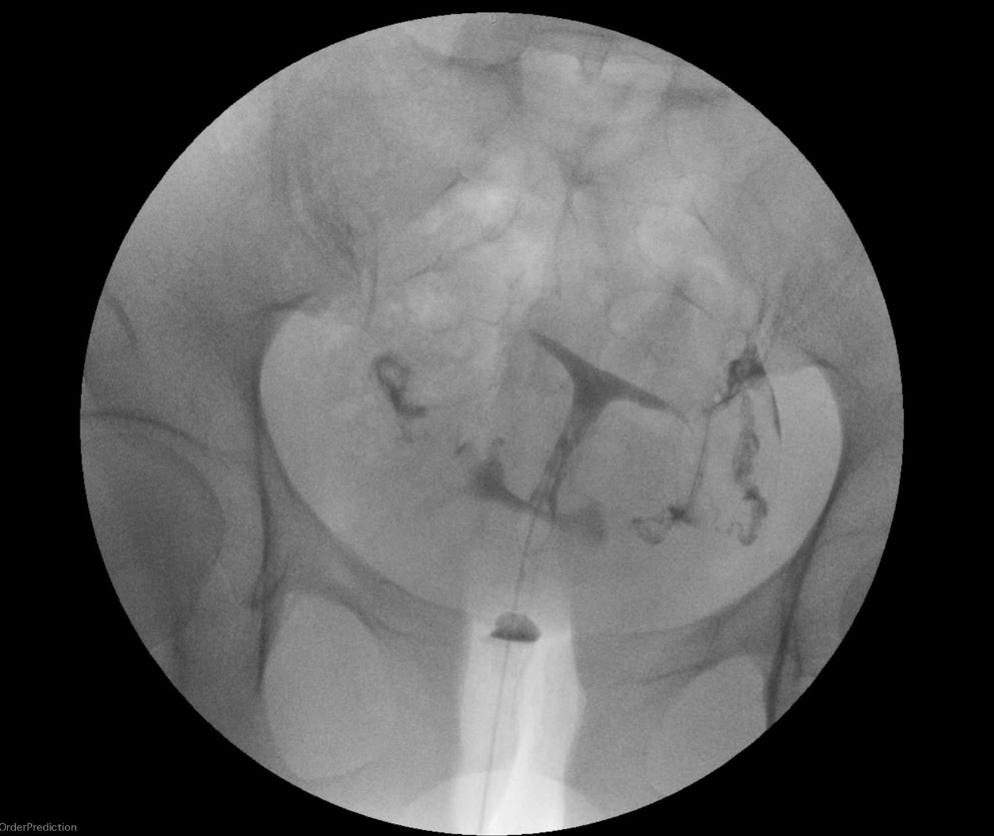
Hysterosalpingography (HSG) of a T‐shaped uterus. Fluoroscopic image acquired during contrast medium injection revealing a tubular endometrial cavity with minimal lateral expansion and a flat or indented fundal contour, consistent with a T‐shaped configuration. Bilateral tubal patency is also observed. This radiographic finding supports the diagnosis of dysmorphic uterus and guides subsequent surgical planning.

The diagnosis of uterine malformations has evolved substantially. Initially, the gold standard involved invasive methods such as combined laparoscopy and hysteroscopy. Over the last 30 years, radiographic tools like hysterosalpingography were widely used for screening, although they lacked precision in evaluating the uterine contour (Figure 1).

**FIGURE 2:**
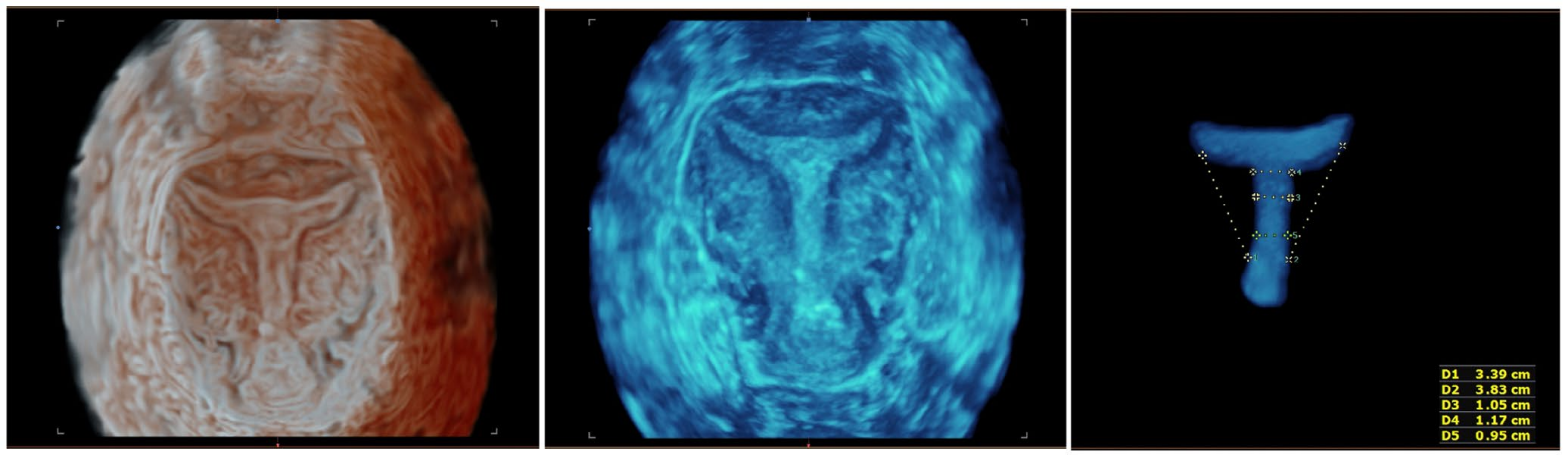
Three‐dimensional (3D) ultrasound of a T‐shaped uterus. Coronal plane reconstructions illustrating a narrow endometrial cavity with thickened lateral walls. (Left, middle) Different rendering modes demonstrate reduced transverse diameter and tubular configuration. (Right) Surface rendering with measurement points confirms a corpus‐to‐cervix ratio consistent with T‐shaped morphology.

The T‐shaped uterus was first classified in the 1988 American Fertility Society (AFS) system, grouping anomalies linked to DES exposure based on subjective imaging interpretation.^3^ For decades, diagnostic approaches relied on clinical judgment without standardized criteria.^12^ The advent of 3D‐TVS significantly improved diagnostic accuracy and is now the reference method due to its non‐invasive nature and high resolution (Figure 2).^12^, ^13^ It allows visualization of the cavity, myometrium, and serosa in the coronal plane. Hysteroscopy also plays a central role by offering both direct visualization and the potential for simultaneous treatment.^14^ Less commonly used modalities include contrast‐enhanced sonohysterography, hysterosalpingography, and magnetic resonance imaging.^12^, ^13^, ^15^ A recent study reported near 100% sensitivity and specificity for 3D‐TVS versus hysteroscopy/laparoscopy in identifying T‐shaped uterus.^16^ However, accurate diagnosis requires specific training and familiarity with updated criteria.^6^, ^17^

Despite growing use, there is still no universally accepted diagnostic standard. At least seven studies have proposed different criteria for T‐shaped uterus.^6^, ^18^, ^19^, ^20^, ^21^, ^22^, ^23^, ^24^ According to the ESHRE/ESGE classification, the anomaly is defined by a narrow cavity with thickened lateral walls but lacks precise cutoffs for wall thickness or cavity width.^1^

In 2015, Exacoustos et al. proposed a 3D‐TVS‐based model requiring two out of three criteria: lateral indentation angle ≤140°, lateral bulging ≥5 mm, and a fundal/isthmic cavity width ratio ≥5.^22^ The Congenital Uterine Malformation by Experts (CUME) group (2019) introduced three coronal plane parameters: indentation depth ≥7 mm, indentation angle ≤130°, and T‐angle ≤40°. Meeting all three confirms diagnosis; two suggests borderline findings.^6^ Pacheco et al. proposed a simplified “rule of 10,” measuring transverse intracavitary diameter 10 mm below the interostial line (R10).^25^

Monaco et al. compared existing models in 50 confirmed cases using 3D‐TVS and hysteroscopy. Their new criteria (lateral bulging ≥5 mm, fundal/isthmic ratio ≥5, and R10 ≤ 10 mm) achieved the highest diagnostic accuracy (area under the curve 0.980), outperforming CUME, which detected only 8% of cases.^8^

Some authors have proposed subclassifications (e.g., T‐, Y‐, I‐shaped uterus), but these are based on subjective interpretation and lack validation.^26^, ^27^ Despite diagnostic advances, robust data on optimal treatment timing and patient selection are still lacking.^18^

**FIGURE 3:**
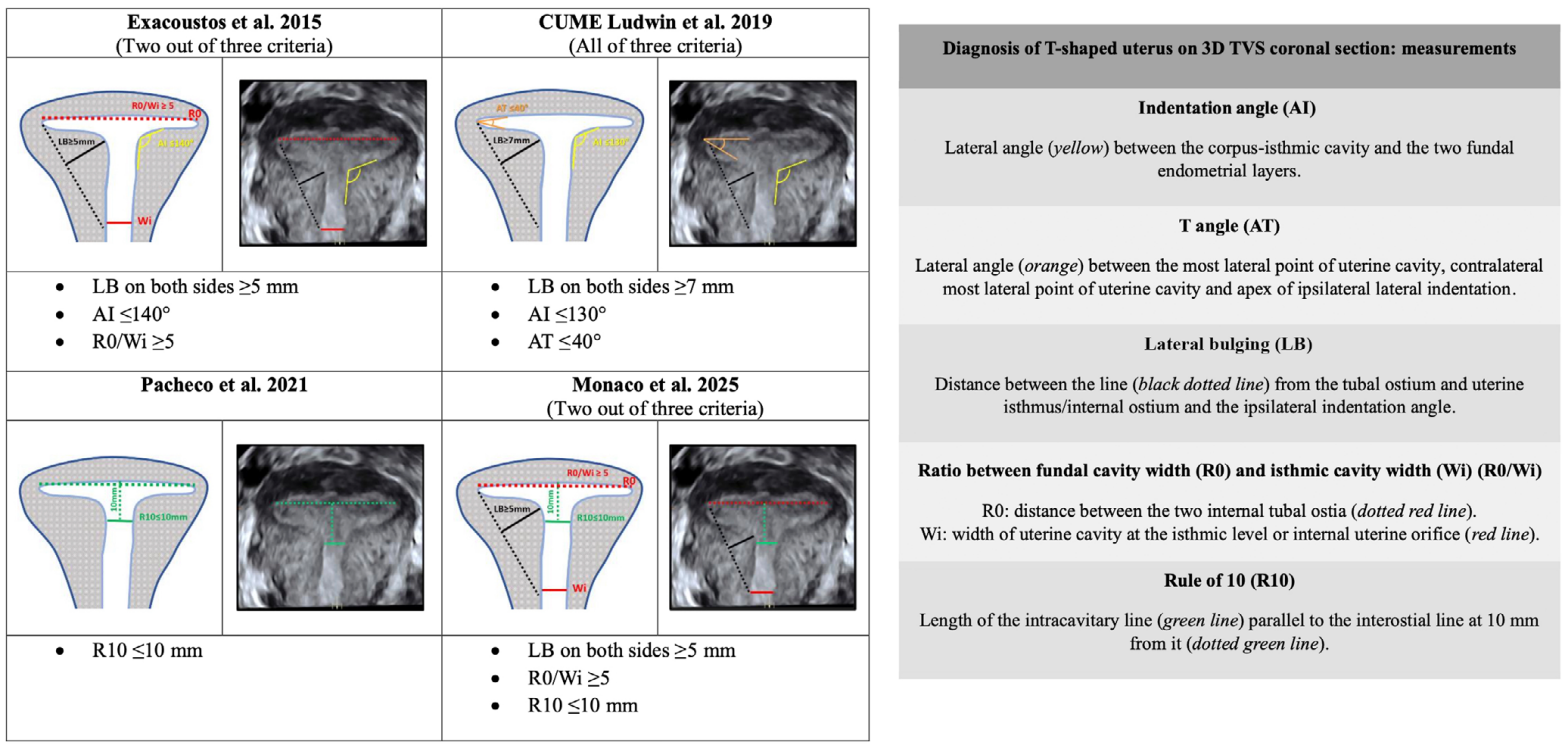
Diagnostic criteria proposed for the identification of T‐shaped uterus. Based on three‐dimensional (3D) transvaginal ultrasound image, these criteria differ in anatomical parameters and diagnostic thresholds.

A 2021 systematic review estimated T‐shaped uterus prevalence at 0.2%–10%, reflecting inconsistencies in criteria and operator expertise.^12^ Determining true prevalence remains difficult, especially since uterine anomalies are rarely assessed in fertile or asymptomatic women.^7^, ^28^ Not initially recognized by ASRM,^29^ heterogeneity across studies stems from different classification systems such as ESHRE/ESGE^1^ and CUME.^12^ Additionally, most available data come from subfertile populations, potentially skewing prevalence estimates and limiting generalizability. Figure 3 provides an overview of the diagnostic criteria used to identify a T‐shaped uterus.

## 4 TREATMENT OF T‐SHAPED UTERUS: UTERINE METROPLASTY

**FIGURE 4:**
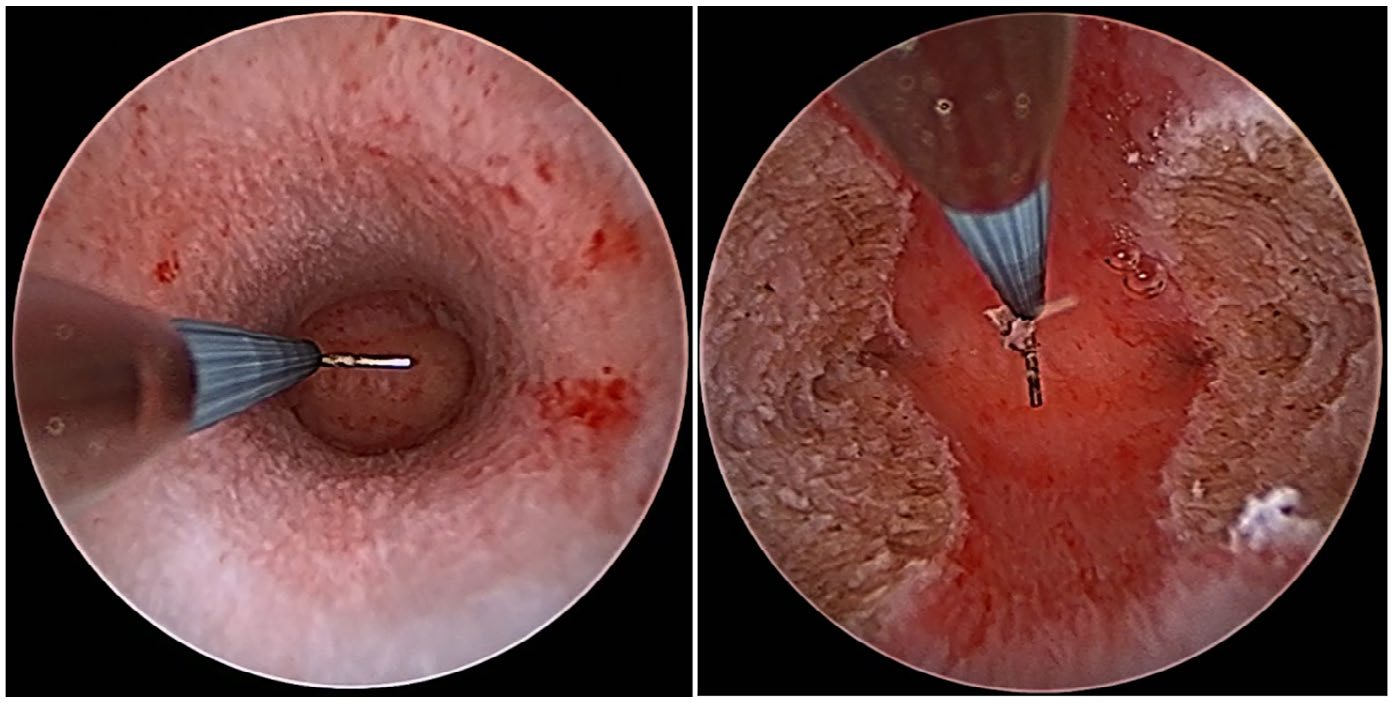
Endoscopic views during hysteroscopic metroplasty. (Left) Narrow tubular endometrial cavity before resection, with thickened lateral walls. (Right) Lateral wall incision performed using a resectoscopic instrument to enlarge the cavity and restore a triangular configuration.

Hysteroscopic metroplasty is considered the treatment of choice for certain Müllerian anomalies, including the T‐shaped uterus (Table S1). The procedure aims to restore a triangular, symmetrical endometrial cavity by resecting excessive myometrial tissue, typically during the follicular phase to optimize visualization (Figure 4).^20^, ^30^, ^31^ Several studies suggest that metroplasty can significantly improve spontaneous pregnancy rates in women with infertility or RPL.^10^, ^30^, ^32^

The conventional technique involves hysteroscopic incisions of the lateral uterine walls, from the tubal ostia to the cervix, using scissors or monopolar electrosurgery. Success is defined by full visualization of the ostia and resolution of subcornual narrowing.^20^, ^31^

Recent innovations have introduced outpatient “see‐and‐treat” techniques using mini‐hysteroscopes under local anesthesia. These approaches reduce procedural time, pain, and cervical trauma, allowing simultaneous diagnosis and treatment.^33^

In 2015, Di Spiezio Sardo et al. introduced the HOME‐DU technique, performed under conscious sedation using a 5‐mm hysteroscope. Incisions target the isthmic rings and anterior/posterior walls using a 5‐Fr bipolar electrode. Postoperatively, anti‐adhesion gel is applied, and follow‐up includes second‐look hysteroscopy and 3D ultrasound, confirming significant cavity volume increase.^34^

In 2021, Catena et al. described an office‐based metroplasty using a 15‐Fr bipolar microresectoscope. Guided by preoperative 3D‐TVS, lateral fibromuscular walls were resected without complications, and cavity normalization was confirmed on follow‐up.^35^

Laser‐based methods, including YAG and diode lasers, offer alternative energy sources. A 2022 retrospective study reported diode laser metroplasty in 25 women with infertility, RIF, or RPL, with no complications and satisfactory postoperative outcomes.^36^, ^37^ Whether improved outcomes result from cavity enlargement or correction of transverse dimensions remains a subject of debated. Di Spiezio Sardo et al. proposed that normalization of lateral width might play a key role in enhancing receptivity.^38^

Intraoperative integration of ultrasound improves anatomical visualization and might reduce the risk of perforation. To prevent adhesions, application of hyaluronic acid‐based or polymeric gels is common practice, although data on their specific efficacy post‐metroplasty remain limited.^34^, ^39^, ^40^ Moreover, sequential estrogen‐progesterone therapy might be prescribed postoperatively to support endometrial regeneration, although evidence on its efficacy is inconclusive.^30^, ^41^

Complication rates after metroplasty are low. Uterine perforation and infection each occur in <1% of cases in expert hands. Moreover, the risk of synechiae or tubal angle adhesions (especially relevant in procedures extending toward the tubal ostia) should be monitored closely.^21^, ^27^ A recent meta‐analysis reported ectopic pregnancy and preterm delivery rates of 4.6% and 12.8%, respectively.^10^ Ducellier et al. observed cervical incompetence in 19% of post‐metroplasty pregnancies, with cerclage needed in eight cases. However, most cases in this study were related to DES. Additionally, the results were not clearly linked to adverse outcomes, and cervical insufficiency is frequently associated with uterine anomalies regardless of surgery.^21^

Overall, both intraoperative (e.g., uterine perforation) and obstetric complications (e.g., cervical insufficiency, ectopic pregnancy) should be taken into account and the procedure reserved for skilled operators. The use of small‐caliber hysteroscopes and early cervical monitoring from 18 weeks of gestation are advisable. Although isolated reports describe cerclage or uterine rupture following metroplasty, robust evidence to quantify these risks is lacking, highlighting the need for further targeted studies.^27^

## 5 INFERTILITY AND REPEATED IMPLANTATION FAILURE IN WOMEN WITH A T‐SHAPED UTERUS

**FIGURE 5:**
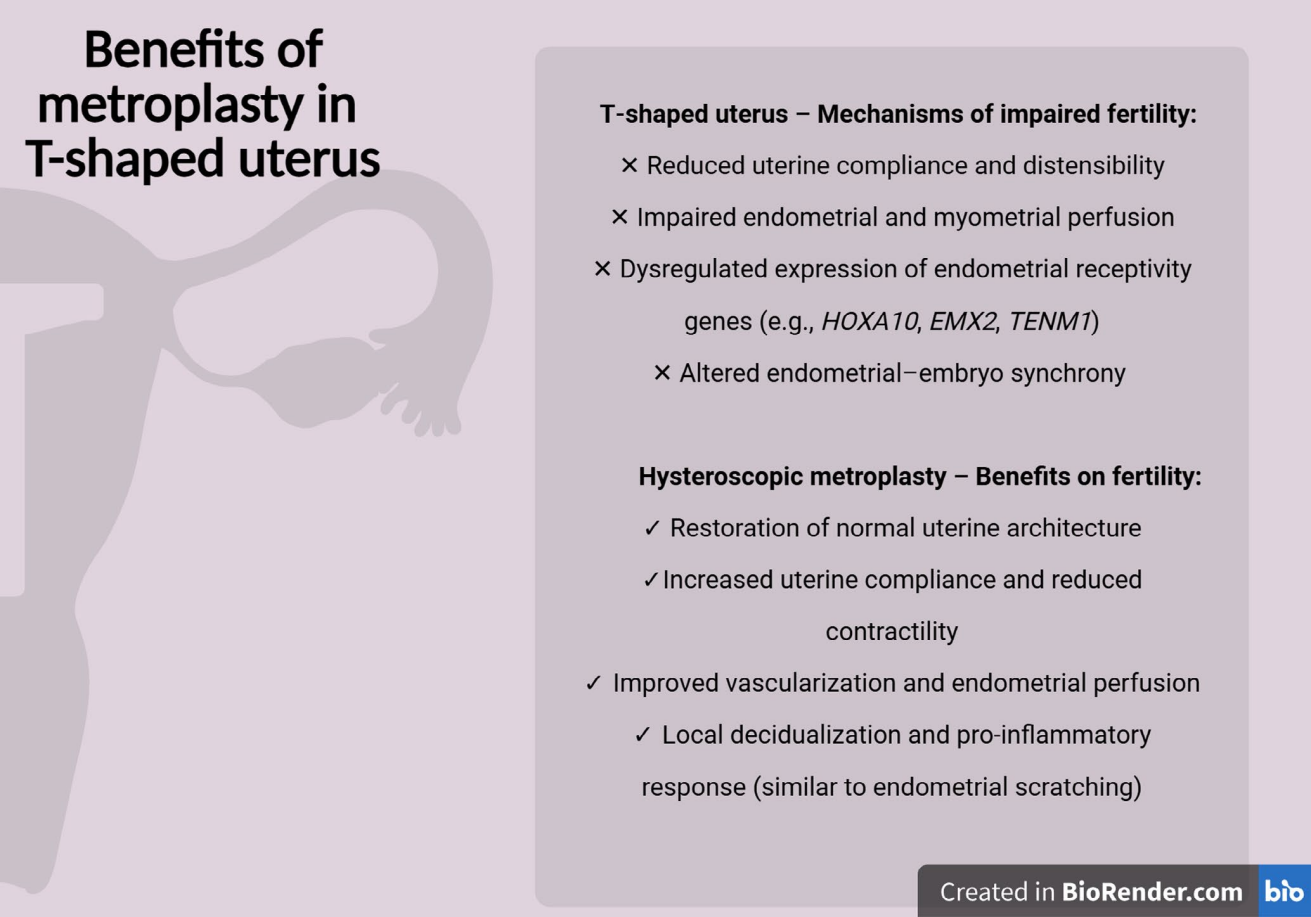
Hypothesized mechanisms underlying reduced fertility in patients with a T‐shaped uterus and the potential reproductive benefits of hysteroscopic metroplasty. The T‐shaped uterus is associated with several factors contributing to impaired fertility, including reduced uterine compliance and distensibility, impaired endometrial and myometrial perfusion, dysregulated expression of endometrial receptivity genes (e.g., HOXA10, EMX2, and TENM1), and altered endometrial–embryo synchrony. Hysteroscopic metroplasty, aimed at restoring a more physiological uterine cavity, might improve fertility by normalizing uterine architecture, increasing compliance and reducing contractility, enhancing vascularization and perfusion, and triggering a local decidual and pro‐inflammatory response, akin to the effects of endometrial scratching.

Müllerian anomalies, particularly a T‐shaped uterus, might create a suboptimal environment for embryo implantation, potentially contributing to infertility and RIF in both natural conception and IVF cycles.^42^ If confirmed by further evidence, surgical correction could enhance implantation rates after fresh or frozen embryo transfers (Figure 5).^43^ The latest ESHRE guidelines define RIF as failure to achieve pregnancy after the transfer of viable embryos across multiple IVF cycles, warranting further evaluation or intervention.^44^ Modern predictive tools incorporate infertility duration, patient characteristics, stimulation protocols, previous IVF history, and embryo ploidy. ESHRE therefore advocates for a personalized approach to RIF diagnosis and management.^44^, ^45^

Accurate patient selection based on standardized diagnostic criteria is essential. Reproductive medicine is particularly prone to overdiagnosis and overtreatment, often influenced by psychological pressures affecting both patients and clinicians. For this reason, surgical intervention in unexplained infertility must be supported by a clear clinical rationale.^46^

The underlying mechanisms of infertility and RPL in women with a T‐shaped uterus remain incompletely understood. Metroplasty might improve outcomes by increasing uterine compliance, enhancing distensibility, and reducing myometrial contractility.^47^ Incisions might also alleviate local vascular constriction, stimulate neovascularization, and enhance perfusion of the endometrium and utero–placental interface.^48^

An additional hypothesis likens metroplasty to endometrial scratching, suggesting that both might promote decidualization or pro‐inflammatory signaling, potentially improving endometrial–embryo synchrony during fresh transfers.^49^ Further, altered gene expression (e.g., HOXA10, EMX2, and TENM1) has been observed in the mid‐secretory endometrium of infertile women with Müllerian anomalies, including septate uterus, compared to fertile controls. HOX genes regulate embryonic morphogenesis and cellular differentiation, and their dysregulation might underlie both anatomical abnormalities and impaired implantation.^50^

## 6 T‐SHAPED UTERUS TREATMENT BEFORE IN VITRO FERTILIZATION

Deciding whether to perform metroplasty in patients with a T‐shape uterus already undergoing IVF, as well as determining the optimal timing for such intervention, remains challenging based on current evidence. Many studies do not adequately account for coexisting causes of infertility in the treated population and often fail to fully consider critical factors such as maternal age and ovarian reserve. Moreover, these studies frequently recommend attempting natural conception after metroplasty and only proceeding to IVF if spontaneous pregnancy does not occur within a defined period.

Among the available studies evaluating metroplasty in women with a T‐shaped uterus, 11 have reported pregnancy outcomes following IVF treatments after surgery, with publications spanning from 2019 to 2024.

The largest available study is a 2020 multicenter retrospective investigation assessing long‐term reproductive outcomes after metroplasty in women with a T‐shaped uterus. Among 214 patients, 166 (77.6%) were diagnosed with primary unexplained infertility, and 48 (22.4%) had a history of early RPL. After a 60‐month follow‐up, the overall clinical pregnancy rate was 72.9% (156/214), and the live birth rate was 80.1% (125/156). Of the 156 women who conceived, 74 (47.4%) did so spontaneously within a median time of 5.5 months, while 82 pregnancies resulted from IVF. Interestingly, 32.4% of those who conceived spontaneously had previously experienced failed IVF or intracytoplasmic sperm injection (ICSI) attempts.^38^

A 2022 retrospective study assessed reproductive outcomes after hysteroscopic metroplasty in 92 women with a T‐shaped (*n* = 30) or Y‐shaped (*n* = 62) uterus and a history of infertility or RPL. Over 2 years, 50 pregnancies (54.3%) were achieved: 56% spontaneous and 44% via assisted reproductive technology. Among IVF pregnancies, eight occurred in T‐shaped and 14 in Y‐shaped uterus cases. Overall, 76% of pregnancies resulted in live births, while 16% ended in miscarriage and 8% in ectopic pregnancy. The study did not distinguish whether adverse outcomes were linked to IVF or natural conception, nor whether IVF followed failed spontaneous attempts.^27^

A prospective observational study (2015–2018) evaluated 111 women with unexplained infertility or ≥2 failed IVF cycles and a diagnosis of T‐shaped (*n* = 48) or septate uterus (*n* = 63) who underwent hysteroscopic metroplasty. After up to 12 months of attempting natural conception, 50% of T‐shaped and 46% of septate uterus patients conceived spontaneously. Among those who proceeded to IVF/ICSI, clinical pregnancy rates were approximately 55% in both groups. No cases of uterine rupture or cervical incompetence were reported. However, the relative contribution of surgery versus IVF remains unclear.^32^

A 14‐year monocentric French study (1992–2016) evaluated 112 women with a T‐shaped uterus. The mean age was 33.2 ± 4.4 years, with an average conception attempt duration of 49.1 ± 32.8 months. Postoperative management included sequential hormonal therapy: initially ethinyl estradiol and lynestrenol, later replaced by estradiol and micronized progesterone. Following surgery, 100 pregnancies were achieved. Among women with primary infertility, 24 conceived via IVF (49%), including one twin pregnancy. IVF outcomes in the RPL subgroup were not reported. Intraoperative complications were minimal (0.9% cervical laceration and false passage), with no uterine perforations. Postoperatively, synechiae occurred in 2.7% of cases, and cerclage was required in eight pregnancies.^21^

A retrospective study of 101 women undergoing hysteroscopic metroplasty for a T‐shaped uterus (monopolar hook needle, followed by estrogen–progesterone therapy) compared IVF outcomes with 148 age‐matched controls with unexplained infertility. Despite a lower delivery rate beyond 20 weeks and more previous IVF failures in the metroplasty group, implantation, clinical pregnancy, and live birth rates after the first embryo transfer were comparable between groups. Endometrial thickness was reduced but within the normal range, and miscarriage rates did not differ. Notably, biochemical pregnancy was higher after fresh transfers in the metroplasty group. Overall, IVF prognosis was similar, but spontaneous conception remained reduced, highlighting the T‐shaped uterus as a relevant infertility factor.^31^

A 2022 prospective cohort of 182 women with a T‐shaped uterus (infertility, RIF, or RPL) confirmed the potential benefit of metroplasty. In women with primary infertility, 32.4% achieved spontaneous pregnancy, with 25.7% live births, while 50.9% of IVF patients conceived, resulting in 38.1% live births. Among those with RPL, 36.6% conceived spontaneously and 30% achieved live births; IVF yielded a 66.6% live birth rate. In the RIF subgroup, no spontaneous pregnancies occurred, but IVF achieved a 39.5% live birth rate. Overall, 37% of women achieved live birth through IVF, indicating that metroplasty might improve reproductive outcomes, particularly in women with RPL or RIF.^42^

Alonso Pacheco et al. conducted a prospective cohort study on 63 nulliparous women with a T‐shaped uterus treated by hysteroscopic metroplasty using 5‐Fr scissors and no electrosurgery, followed by 3 months of estrogen–progestin therapy. Of the 60 women attempting conception, 50 became pregnant, yielding an overall clinical pregnancy rate of 83.3% and a live birth rate of 76% (38/50), both significantly improved after surgery (*P* < 0.001). Fifteen pregnancies were spontaneous (11 live births), while 35 occurred via IVF (27 live births). In the expectant group, 35.9% conceived spontaneously. Among women with RPL, the miscarriage rate dropped to 16.7% (*P* < 0.001).^20^

A study evaluated IVF outcomes in 90 women with a T‐shaped uterus and no other identifiable infertility factors, all diagnosed with RIF (≥3 failed embryo transfers). In this cohort, 48 patients underwent hysteroscopic metroplasty followed by a 3‐month attempt at spontaneous conception, after which IVF was initiated if needed. The remaining 42 patients received IVF without prior surgical intervention. After metroplasty, five patients conceived spontaneously, reducing the population for IVF comparison to 85 women. In the metroplasty + IVF group (*n* = 43), 24 achieved pregnancy, corresponding to an implantation rate of 55.8%, and a live birth rate of 30.2% (13/43). In contrast, in the IVF‐only group (*n* = 42), only 11 pregnancies occurred (26.2% implantation rate), with just four resulting in live births (9.5%).^51^

The optimal timing of conception following metroplasty was explored in a retrospective study, which set the primary endpoint as the occurrence of a live birth within 18 months after surgery. The study involved 43 nulliparous women with an average duration of primary infertility of 5.2 ± 2.4 years. Notably, 84.2% had undergone at least one IVF cycle before the metroplasty. At 18 months post‐surgery, 12 pregnancies were recorded (27.9% live birth rate), evenly split between spontaneous and IVF conceptions. Over a median follow‐up of 4.5 years, 53.5% of women achieved pregnancy, although it was not specified whether these pregnancies were spontaneous or assisted. These findings suggest that a substantial proportion of pregnancies occur within the first 18 months after metroplasty, emphasizing the importance of timely intervention to mitigate the age‐related decline in fertility.^52^

Advances in hysteroscopic techniques have enabled outcome analysis based on the specific surgical modality employed. In a 2022 study, Bylgory et al. examined reproductive outcomes following diode laser hysteroscopic metroplasty in women with dysmorphic uteri, including T‐shaped and Y‐shaped anomalies. Postoperatively, patients were treated with estradiol and medroxyprogesterone acetate hemihydrate for 2 months. Of 25 women who underwent diode laser metroplasty, 22 attempted conception: one conceived spontaneously and 12 via IVF, confirming the technique's safety and effectiveness in nulliparous women with a dysmorphic uterus.^36^

A complementary study conducted in 2020 evaluated pregnancy outcomes following the DU‐HOME metroplasty technique in 63 women with dysmorphic uteri: mostly T‐shaped, with a minority of Y‐shaped and I‐shaped forms. Over a follow‐up ranging from 34 to 195 weeks, 48 women achieved pregnancy (76.2%), and 36 delivered live births (64.3%). Among infertile patients, 18 pregnancies resulted in live births: six spontaneous (33.3%), eight via intrauterine insemination (44.4%), and four via IVF (22.2%). In the RPL subgroup, 18 live births were recorded: seven spontaneous (38.9%), three via intrauterine insemination (IUI), and eight via IVF (44.4%).^53^

## 7 DISCUSSION

Over the past 10–15 years, the diagnosis and treatment of the T‐shaped uterus have gained increasing importance in the field of reproductive medicine. While previous reviews have primarily focused on outcomes related to spontaneous conception, our aim was to investigate how hysteroscopic metroplasty might influence outcomes in the context of IVF. In some of the reviewed studies, after a variable period of attempting spontaneous conception, the percentage of women who achieved pregnancy through IVF even exceeded those who benefited from hysteroscopic metroplasty alone in terms of restoring natural fertility.^20^, ^36^, ^42^

**FIGURE 6:**
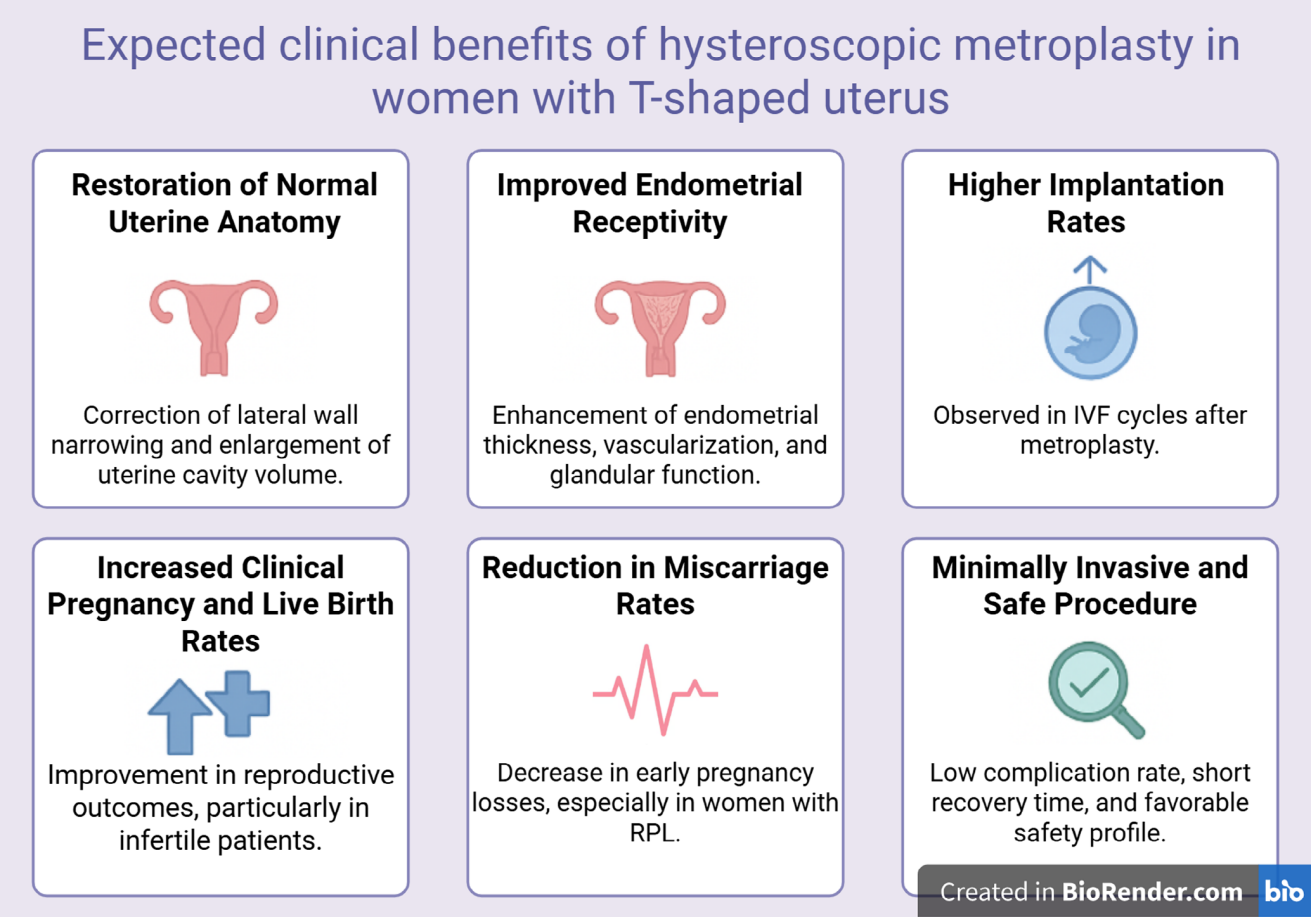
Schematization of the potential clinical benefits observed following hysteroscopic metroplasty in patients with T‐shaped uterus.

The studies included in this review exhibit substantial heterogeneity in design, patient populations, and outcome reporting. Nevertheless, across all included studies, a proportion of patients successfully conceived through IVF.^20^, ^21^, ^27^, ^31^, ^32^, ^36^, ^38^, ^42^, ^51^, ^52^, ^53^ This finding is particularly relevant for women with repeated IVF failures (Figure 6), in whom metroplasty might offer a targeted correction of uterine dysmorphism and improve endometrial receptivity. However, most studies provide limited details on IVF protocols, such as the use of conventional IVF versus ICSI, embryo stage at transfer (cleavage vs. blastocyst), or whether fresh or frozen cycles were used. The potential role of endometrial preparation with estrogen in optimizing receptivity also remains underexplored. Moreover, focusing solely on the first embryo transfer might underestimate the cumulative impact of treatment.^31^

Several key questions remain regarding the reproductive benefit of metroplasty in women with a T‐shaped uterus. If infertility is primarily uterine in origin, surgical correction should theoretically restore natural fertility.^27^ Yet, available data do not clearly support this assumption. One contributing factor might be that many women had already undergone ovarian stimulation and cryopreserved embryos prior to surgery, subsequently proceeding with embryo transfer without attempting spontaneous conception.^27^, ^51^ Randomized controlled trials involving euploid embryo transfers are needed to isolate the effect of uterine factors and more precisely define metroplasty's role (47). Another possible explanation is the frequent underdiagnosis of uterine malformations, particularly those classified in the ESHRE U2 group (including infantile or T‐shaped forms), which are often identified only after fertility treatments have begun.^1^ As a result, a decline in ovarian reserve might occur during unsuccessful treatment cycles carried out prior to metroplasty, ultimately necessitating further therapeutic support in the pursuit of pregnancy.^54^ Even the study by Pabuccu et al.^32^ highlighted this possibility: in couples with unexplained infertility or more than two implantation failures, the clinical pregnancy rate after metroplasty was 50%. Among the remaining women, only 55% achieved pregnancy through IVF after 1 year of unsuccessful attempts at spontaneous conception. Finally, it remains possible that the couples analyzed were affected by infertility of a different etiology, with the uterine anatomical abnormality acting as a contributing, but not primary, factor. In such cases, surgical correction might support implantation, but it is IVF treatment that ultimately proves decisive in achieving pregnancy.

Endometrial growth stimulation during follicular phase through the administration of exogenous estrogens, either orally or transdermally, might also support embryo implantation in women who have undergone metroplasty.^21^ The observation of reduced endometrial growth is further supported by the study conducted by Uyar et al., which showed that women undergoing IVF due to a T‐shaped uterus exhibited significantly lower endometrial thickness compared to those undergoing IVF for other causes of infertility.^31^ Women undergoing IVF might benefit from embryo transfer not in a natural cycle, but rather in a hormonally controlled cycle, in which exogenous endometrial stimulation could promote proper development of the surgically treated endometrial areas. Similar findings have been reported in women with adenomyosis, who appear to benefit from higher doses of progesterone for luteal phase support, likely due to an alteration and dislocation of progesterone receptors at the uterine level.^55^, ^56^

One unresolved issue is whether a trial period of spontaneous conception should be attempted after metroplasty and its duration in women with infertility or RPL or whether immediate progression to IVF is more appropriate. It remains to be clarified whether these women should be given the standard 12‐month period defined in the classic definition of infertility or whether a shorter timeframe might be more appropriate.^52^, ^57^ This decision must be individualized, based on the couple's clinical history and the presence of other infertility factors. If the T‐shaped uterus is identified as the sole cause of infertility, spontaneous conception might be a reasonable first step, particularly in younger patients with preserved ovarian reserve. However, delaying treatment could reduce cumulative pregnancy rates due to advancing maternal age and declining fertility over time.^21^, ^38^, ^52^

## 8 CONCLUSION

The T‐shaped uterus is a structural anomaly linked to reduced fertility, increased risk of RIF, and RPL. Hysteroscopic metroplasty appears to improve reproductive outcomes (especially implantation, clinical pregnancy, and live birth rates) based on observational data. While promising, the evidence is limited by small sample sizes, lack of randomized controlled trials, and heterogeneity in diagnostic and treatment protocols. The procedure might be considered in selected patients with a T‐shaped uterus and repeated IVF failures, particularly after exclusion of other infertility factors. However, the precise mechanisms underlying its benefit remain unclear. High‐quality, standardized prospective studies are urgently needed to clarify its efficacy and optimize patient selection.

## AUTHOR CONTRIBUTIONS

Conceptualization: Simone Ferrero. Formal analysis: Alessandro Favilli, Consuelo Russo and Giulia Monaco. Investigation: Franco Alessandri and Simone Ferrero. Methodology: Fabio Barra and Irene Gazzo. Supervision: Simone Ferrero, Sandro Gerli and Caterina Exacoustos. Writing – original draft: Irene Gazzo and Fabio Barra. Writing – review & editing: Luis Alonso Pacheco and Caterina Exacoustos.

## FUNDING INFORMATION

The manuscript has been not funded.

## CONFLICT OF INTEREST STATEMENT

The authors do not have conflicts of interest to declare.

## Supporting information


Table S1

## Data Availability

Data sharing is not applicable to this article as no new data were created or analyzed in this study.
