# Supplementary material for: Metroplasty before IVF in women with a T‐shaped uterus: A clinical challenge
Source: Int J Gynaecol Obstet. 2026 Jan 22;174(1):86–95. doi: 10.1002/ijgo.70827 (PMC13278672; doi:10.1002/ijgo.70827)
Supplement: Supplementary file 1 — Table S1 [file IJGO-174-86-s001.docx]

**Supplementary Table 1.** Surgical techniques proposed for treating T-shaped uterus

| **Author / Study** | **Year** | **Technique** | **Instrument** | **Setting** | **Adjuncts / Follow-up** | **Key Findings** |
| --- | --- | --- | --- | --- | --- | --- |
| Unspecified (traditional technique) [14] | * | Standard metroplasty | Monopolar hook or bipolar scissors | OR setting | Sequential estradiol (E2) + progesterone (P4) therapy | Low complication rate (<1%), widely adopted |
| Di Spiezio Sardo et al. [34] | 2015 | HOME-DU technique | 5-mm hysteroscope + 5-Fr bipolar electrode | Outpatient (conscious sedation) | Anti-adhesion gel + 3D-TVS + second-look hysteroscopy | Increased cavity volume; effective and minimally invasive |
| Catena et al. [35] | 2021 | Lateral fibromuscular wall resection | 15-Fr bipolar mini-resectoscope | Office-based | Guided by pre-op 3D-TVS | Cavity normalization, no complications |
